# The impact of COVID-19 on referrals among general practitioners and specialists in Shanghai, China

**DOI:** 10.1017/S1463423624000525

**Published:** 2024-11-20

**Authors:** Zhongqing Xu, Jingchun Fan, Dandan Shi, Jingjing Ding, Jun Zhou, Xianzhen Feng, Brett D. Hambly, Kun Tao, Shisan Bao

**Affiliations:** 1 Department of General Practice, Tongren Hospital, Shanghai Jiao Tong University, School of Medicine, Shanghai, China; 2 Discipline of General Practice, Shanghai Jiao Tong University, School of Medicine, Shanghai, China; 3 Center for Community Health Care, China Hospital Development Institute, Shanghai Jiao Tong University, Shanghai, China; 4 School of Public Health, Gansu University of Chinese Medicine, Lanzhou, China; 5 Center for Evidence-Based Medicine, Gansu University of Chinese Medicine, Lanzhou, China

**Keywords:** COVID-19, GP, referral, specialists

## Abstract

**Background:**

The COVID-19 pandemic has impacted patient’s visits to general practitioners (GPs). However, it is unclear what the impact of COVID-19 has been on the interaction among the local primary care clinics, the GP Department within the hospital and specialists.

**Methods:**

The interaction among GPs referring to hospital-based specialists and specialists to local doctors was determined, comparing pre-pandemic 2019 and 2020 during the pandemic.

**Results:**

Reduced referrals from GPs to specialists were consistent with the reduction in specialist referrals back to the local doctors, which dropped by approximately 50% in 2020, particularly in the two most common chronic conditions (hypertension and diabetes mellitus).

**Discussion:**

Reduced referral of patients from local clinics to Tongren Hospital is probably due to the extensive online training provided to the local GPs to become more competent in handling local patients via telehealth. Our data provide some insight to assist in combatting the pandemic of COVID-19, offering objective evidence of the impact of COVID-19 on patient management by GPs.

## Introduction

General practitioners (GPs) have played a critical role in managing/limiting transmission of COVID-19 (Liu *et al.*, 2020) since the beginning of the pandemic (Cao *et al.*, 2020). The role of GPs in China is rather unique since GPs practice as specialists in the GP Department within Secondary/Tertiary hospitals, in addition to conducting community clinics. Thus, complex patients with an uncertain diagnosis and/or serious conditions are referred from the community clinics to the GP Department within hospitals; whereas patients with a clear diagnosis and management plan are transferred back to the community clinics for routine outpatient management. Very complex patients can also be referred beyond the GP Department to specialists in specific disciplines, such as internal medicine and surgery.

During the COVID-19 outbreak in 2020, the GP college in Shanghai was able to provide tailored online educational courses to meet the needs of local community doctors, designed to facilitate the implementation of proper handling procedures for patients suspected of COVID-19 infection (Shi *et al.*, 2020). Such online targeted training boosted self-confidence, which has enabled local doctors to manage these patients more effectively in local community clinics, including retaining many of these patients in the local communities during COVID-19 outbreaks. Overall, during the pandemic, there have been substantial changes in medical practice in most areas. To minimize/reduce potential viral transmission, many patients voluntarily reduced face-to-face visits in the GP Department (Xu *et al.*, 2021) and instead chose Internet-based consultations. Even when these patients had to come to the GP Department, they opted for larger prescriptions to minimize the frequency of hospital visits, particularly among those with quite stable chronic conditions. Our GPs were conscious of the potential risks of larger prescriptions reducing the interval between patient reviews. However, both the patients and doctors were coping with such challenges, and the outcomes were proven to be very successful.

Tongren Hospital is the only general hospital in the Changning region, supervising 10 community clinics in this region, and manages the health of nearly 700 000 people. However, it is unclear whether the pandemic during 2020 had any significant impact on GPs, particularly in relation to changed patterns of referral of patients to or from local community clinics to the GP Department of Tongren Teaching Hospital, and from the GP Department to other Discipline-specific specialists in Tongren Hospital. The aim of this study was to understand the management of patients in the Department of GP, including the referral process to different specialized departments during the most challenging event of the century, i.e., the COVID-19 pandemic.

## Materials and methods

### Study design

A retrospective investigation was conducted in Tongren Hospital, Shanghai from January 2019 to December 2020. This study was approved by the Medical Ethics Committee of the hospital (NO. 2020-079-01) and strictly followed the World Medical Association’s Declaration of Helsinki. All the de-identified data obtained were from the electronic health record system of Tongren Hospital. These patients were requested to sign consent forms for the use of their data for medical research, in addition to medical treatments, during their routine initial registration. Therefore, we had access to these data prior to, during, and post-COVID-19 periods.

Data that were collected were from the Centre database and included all the patient data for patients who visited GP outpatient clinics in person and were transferred from community clinics to Tongren Hospital during the entire year of 2020, as well as the corresponding period in 2019. We measured patients’ age, sex, number, and proportion referred from the community to the hospital GP Department or from the hospital GP Department to the community, particularly those with hypertension and diabetes. Additionally, we measured the number and proportion of patients referred from GP Department to specialists in specific Disciplines or from specialists in specific Disciplines to the community.

### Statistical analysis

Quantitative variables were expressed as means with standard deviation (SD), and qualitative variables were expressed as percentages. Differences were evaluated using the Chi-square test for categorical variables, and *t* test for normally distributed variables and the Mann-Whitney *U* nonparametric test for non-normally distributed variables after consulting with a medical statistician.

## Results

### Community referrals of patients to the GP department, Tongren hospital

**Figure 1.**
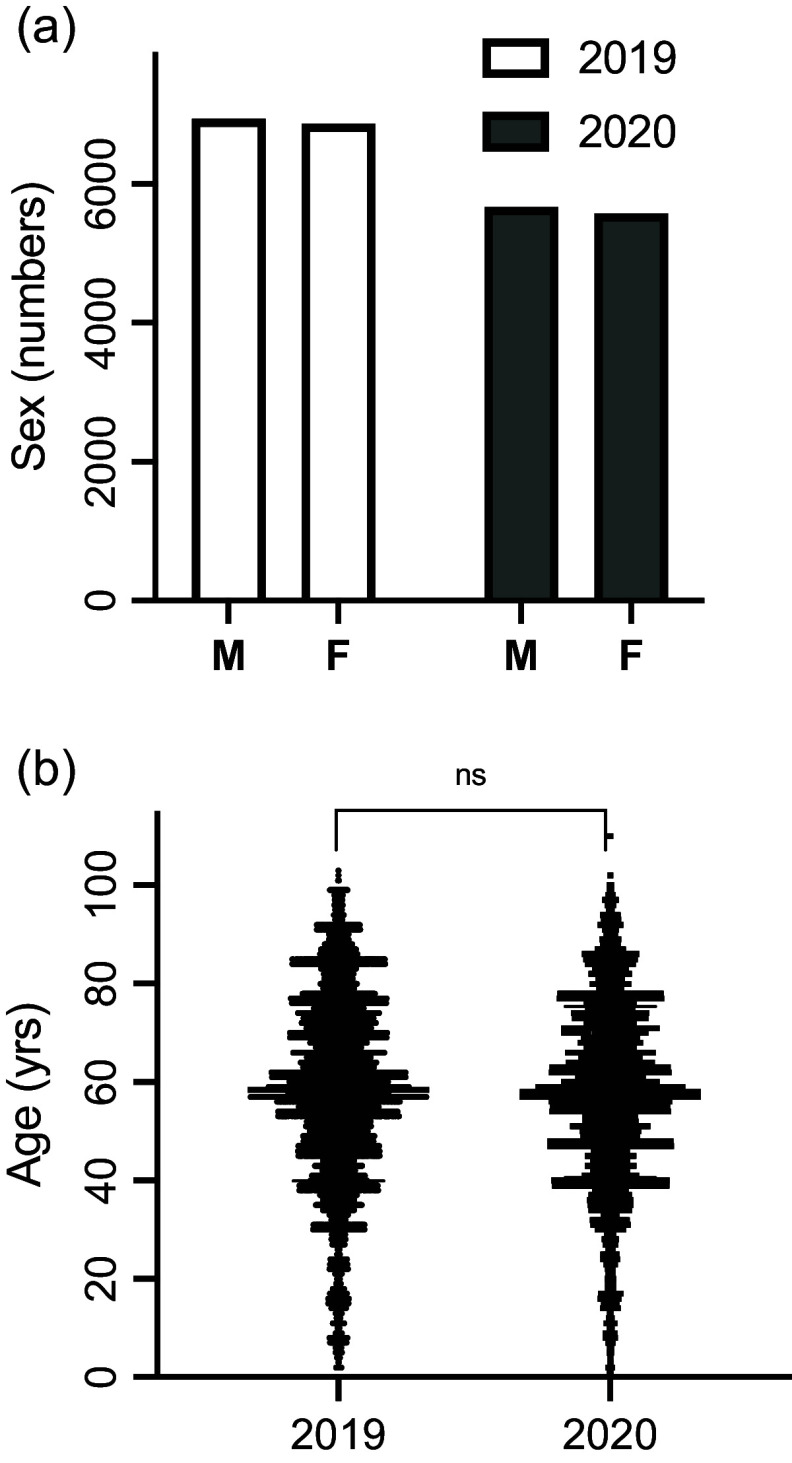
The number of patient referrals to the Department of GP, Tongren Hospital, in the years 2019 and 2020. The X-axis represents the male vs female numbers (a), and the Y-axis represents the number of patient referrals. The age distribution of the patients’ visits in 2019 and 2020 is also shown (b). n.s.: statistically not significant.

**Figure 2.**
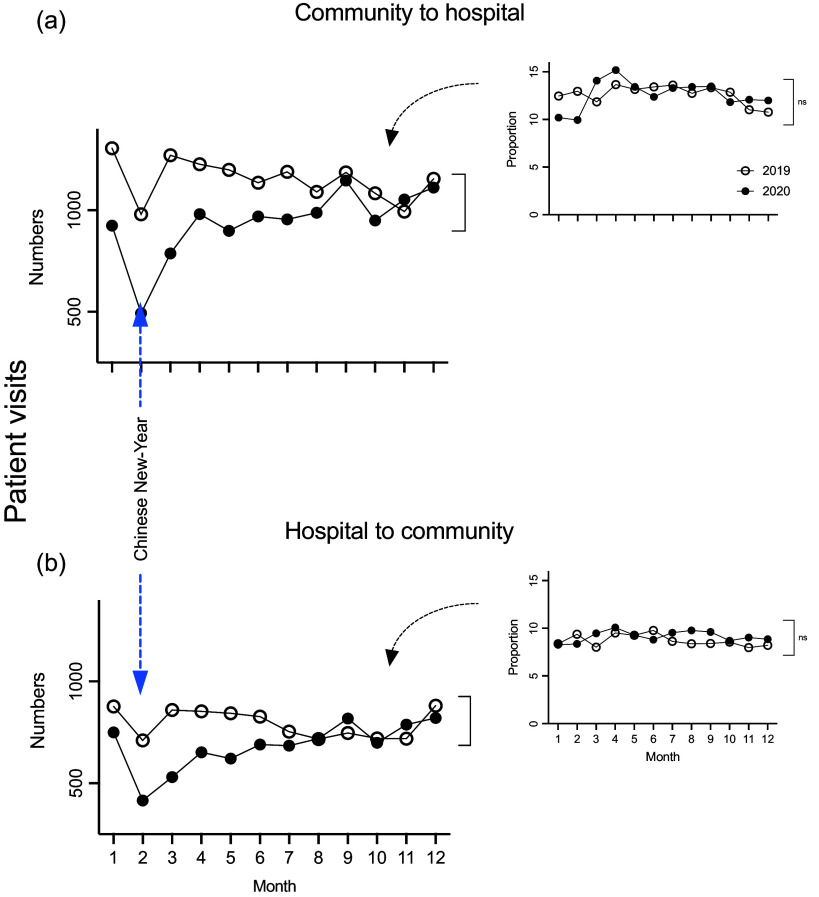
The number of patients referred from the local clinics to the Department of GP, Tongren Hospital, showing the monthly distribution in years 2019 and 2020 (a), and the patients referred back from the Department of GP, Tongren Hospital (b). The Y-axis represents the number of patient referrals. The insets show the number of patient referrals to the hospital GP clinic each month as a proportion of total numbers of patients seen within the GP community clinics each month, as a percentage. n.s.: statistically not significant.

Overall, there was a 16% drop in the number of patients transferred from community clinics to the GP Department, Tongren Hospital in 2020, compared to 2019 (11 253 *vs* 13 809), of which 5,580 (49.6%) or 6,870 (49.8%) were female in 2020 or 2019, respectively (Table 1, Figure 1a, *P* > 0.05). There was no significant difference in the average age of the patients between 2020 and 2019 (57.99 ± 17.42 *vs* 58.43 ± 18.51 yr) (Table 1, Figure 1b, *P* > 0.05). On a monthly basis, the decrease in patient referrals was maximal in February/March 2020 at approximately 40%–50%, with a subsequent slow recovery through to October 2020 (Table 2, Figure 2a, *P* < 0.01). Notably, when the number of referrals was considered as a proportion of the total number of patients seen within the GP Department each month, there was no significant difference in the proportion of patients being referred over the 12-month period in 2020 compared to 2019 (Table 2, Figure 2a, inset, *P* > 0.05).

**Table 1.** Demography of patients’ referral from community to hospital in 2020, compared to corresponding data from 2019

Characteristics	2019	2020	P-value
Total number	13 809	11 253	–
Age, mean ± S D (Yr)	58.43 ± 18.51	57.99 ± 17.42	0.062
Sex, *n* (%)			
Male	6,939 (50.2)	5,673 (50.4)	0.797
Female	6,870 (49.8)	5,580 (49.6)	0.797

**Table 2.** The monthly patients’ number and proportion in community to hospital and hospital to community during 2019 and 2020

	Community to hospital				Hospital to community			
	Numbers		Proportion (%)		Numbers		Proportion (%)	
	2019	2020	2019	2020	2019	2020	2019	2020
Jan	1305	924	12.47	10.19	877	750	8.38	8.27
Feb	980	493	12.95	9.94	710	415	9.38	9.37
Mar	1270	787	11.85	14.07	860	530	8.02	8.48
Apr	1226	981	13.67	15.18	853	652	9.51	10.09
May	1198	898	13.16	13.42	844	621	9.27	9.28
Jun	1135	969	13.42	12.37	827	690	9.78	8.81
Jul	1189	955	13.61	13.30	754	685	8.63	9.64
Aug	1090	987	12.74	13.42	717	719	8.38	9.77
Sep	1186	1146	12.34	13.48	747	818	8.40	9.62
Oct	1083	949	12.86	11.82	720	699	8.55	8.70
Nov	993	1053	11.02	12.08	719	788	7.98	9.04
Dec	1154	1111	10.77	11.99	881	821	8.22	8.86
*P* value	0.001		0.927		0.011		0.079	

### Hospital GP department referrals of patients back to community clinics

The total number of patients transferred from the GP Department at Tongren Hospital back to the community clinics decreased by 11% overall in 2020 compared to that of 2019 (8,188 *vs* 9,509). On a monthly basis, the decrease in patient referrals was maximal in February/March at approximately 40%, with a subsequent slow recovery through to July 2020 (Table 2, Figure 2b, *P* < 0.01). There was no significant difference in the proportion of referrals compared to the total number of patients being seen within the GP Department between 2019 and 2020 (Table 2, Figure 2b, inset, *P* > 0.05).

### Department of GP referrals of patients to specialists in specific disciplines

**Figure 3.**
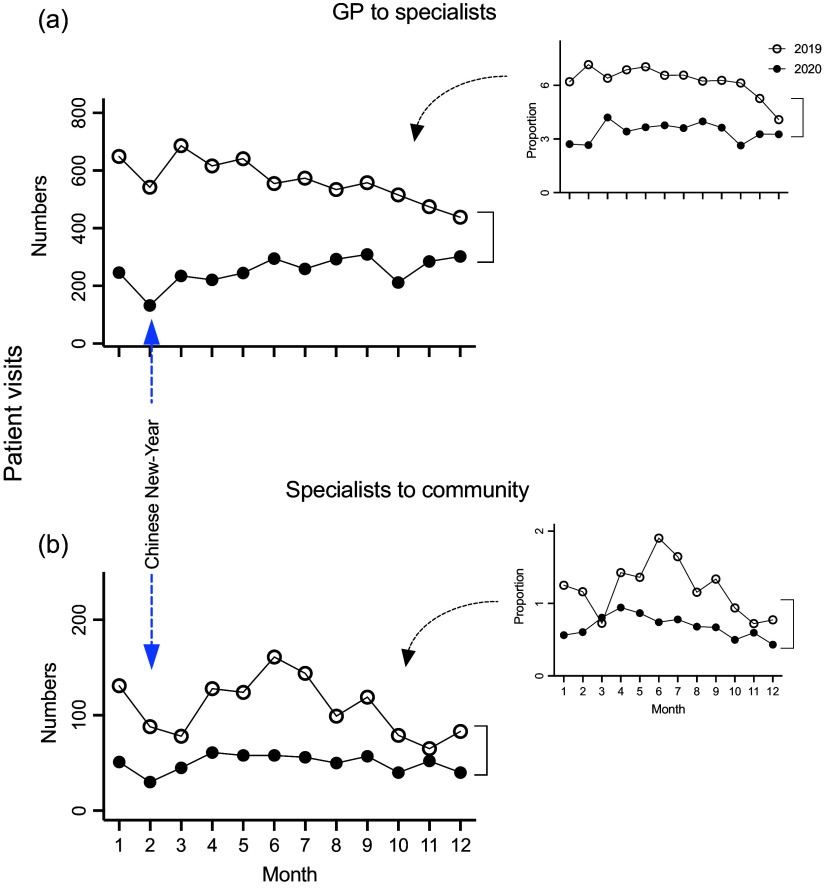
The number of patients referred from the GP Department to the Specialists in Tongren Hospital in the years 2019 and 2020 (a); whereas the patients referred from the specialist back to local clinics are also shown (b). The insets show the number of patient referrals each month to hospital specialists as a proportion of total numbers of patients seen within the GP community clinics each month, as a percentage.

The patient numbers referred from the GP Department to the specific specialists in Tongren Hospital (eg internal medicine or surgery) decreased by ∼55% in 2020 compared to that in 2019 (3,034 *vs* 6,784, respectively). This decrease was large across all of 2020, varying between a 76% drop in February and a 31% drop in December 2020, compared to monthly data in 2019 (Figure 3a, *P* < 0.001). Notably, when the proportion of patients referred to the specialists versus the total number of patients seen in the GP Department was compared between 2019 and 2020, a similar pattern of difference was observed, compared to that of the absolute number, showing substantially reduced referrals from the GPs to specific-discipline specialists (Table 3, Figure 3a, inset, *P* < 0.001).

**Table 3.** The monthly patients’ number and proportion in GP to specialist and specialist to community during 2019 and 2020

	GP to specialist				Specialist to community			
	Numbers		Proportion (%)		Numbers		Proportion (%)	
	2019	2020	2019	2020	2019	2020	2019	2020
Jan	649	246	6.20	2.71	131	51	1.25	0.56
Feb	542	132	7.16	2.66	88	30	1.26	0.60
Mar	686	235	6.40	4.20	78	45	0.73	0.80
Apr	616	221	6.87	3.42	128	61	1.43	0.94
May	641	245	7.04	3.66	124	58	1.36	0.74
Jun	555	295	6.56	3.77	161	58	1.90	0.87
Jul	574	259	6.57	3.61	144	56	1.65	0.78
Aug	534	293	6.24	3.98	99	50	1.16	0.68
Sep	558	309	6.28	3.63	119	57	1.34	0.67
Oct	516	212	6.13	2.64	79	40	0.94	0.50
Nov	475	285	5.27	3.27	65	52	0.72	0.60
Dec	438	302	4.09	3.26	83	40	0.77	0.43
*P* value	< 0.001		< 0.001		< 0.001		< 0.001	

### Specialist referral of patients back to the community clinics

The overall patient numbers that specialists in specific disciplines transferred back to the community clinics decreased by 54% in 2020 compared to that in the same period of 2019 (598 *vs* 1,299, respectively) (Figure 3b, *P* < 0.001). Interestingly, the relative number of referrals was gradually reduced from January to March 2019, then gradually increased from March and peaked in June 2019, and then slowly decreased till December 2019 (Figure 3b). Notably, when the proportion of patients referred back from the specialists versus the total number of patients seen in the GP Department were compared between 2019 and 2020, a similar pattern of difference was observed, compared to that of the absolute number, showing substantially reduced referrals from the specialists back to the GPs (Table 3, Figure 3b, inset, *P* < 0.001).

### Management of classic chronic diseases during the COVID-19 pandemic

**Figure 4.**
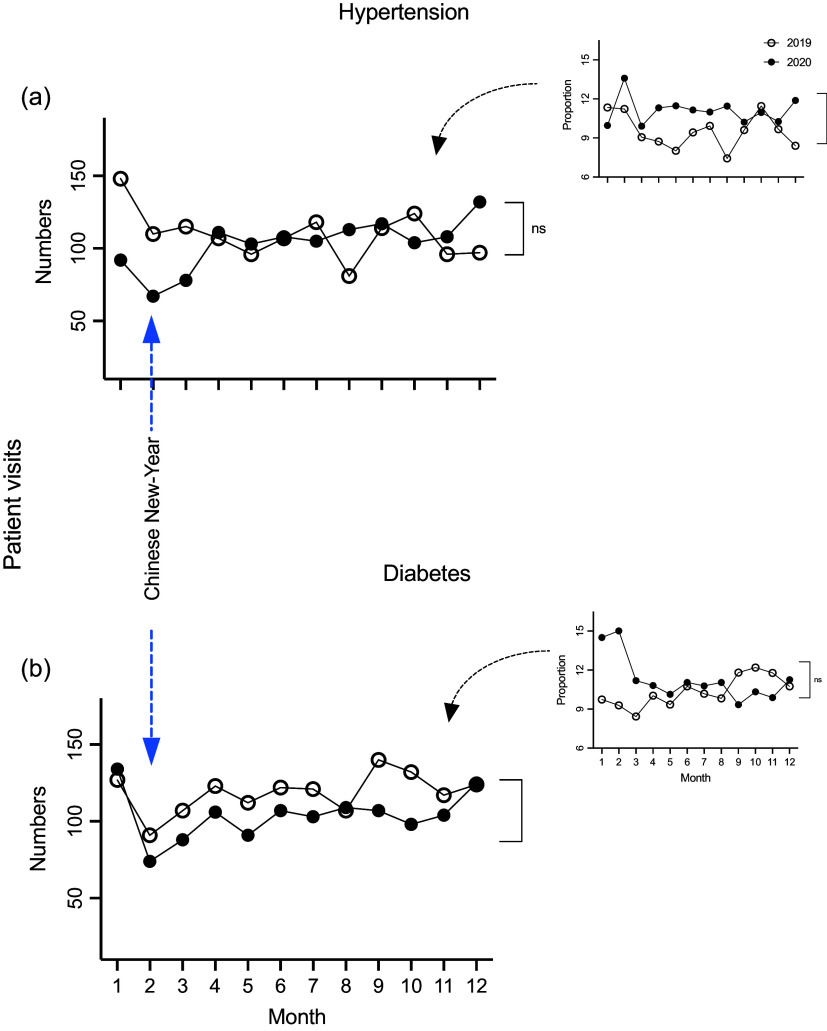
The number of hypertensive patients referred from the local clinics to the Department of GP, Tongren Hospital (a) in the years 2019 and 2020; compared to the number of the diabetes mellitus patients referred to the Department of GP, Tongren Hospital during 2019 and 2020 (b). The insets show the number of hypertensive (Figure 4a inset) or DM (Figure 4b inset) patient referrals each month to the hospital GP clinic as a proportion of total numbers of hypertensive DM patients, respectively, seen within the GP community clinics each month, as a percentage. n.s.: statistically not significant.

An assessment of the impact of COVID-19 on the most common chronic diseases (hypertension and diabetes mellitus [DM]) in China was undertaken for the current study. The total number of hypertension patients referred from community clinics to the GP Department within Tongren Hospital decreased by 6% in 2020, compared to 2019 (1,238 *vs* 1,313, respectively), although this change was not found to be significant (Figure 4a, *P >* 0.05). However, surprisingly, there was a significant increase in the relative number of hypertensive patients referred as a proportion of the total number of hypertensive patients seen by GPs in 2020 compared to 2019 (Table 4, Figure 4a, inset, *P* < 0.01).

**Table 4.** The monthly patients’ number and proportion with chronic diseases in community to hospital during 2019 and 2020

	Hypertension				Diabetes			
	Numbers		Proportion (%)		Numbers		Proportion (%)	
	2019	2020	2019	2020	2019	2020	2019	2020
Jan	148	92	11.34	9.96	127	134	9.73	14.50
Feb	110	67	11.22	13.59	91	74	9.29	15.01
Mar	115	78	9.05	9.91	107	88	8.43	11.18
Apr	107	111	8.73	11.31	123	106	10.03	10.81
May	96	103	8.01	11.47	112	91	9.35	10.13
Jun	107	108	9.43	11.15	122	107	10.75	11.04
Jul	118	105	9.92	10.99	122	103	10.18	10.79
Aug	81	113	7.43	10.45	107	109	9.82	11.04
Sep	114	117	9.61	10.21	140	107	11.80	9.34
Oct	124	104	11.45	10.96	132	98	12.19	10.33
Nov	96	108	9.67	10.27	117	104	11.78	9.88
Dec	97	132	8.41	11.88	104	125	10.75	11.25
*P* value	0.381		0.003		0.020		0.133	

In contrast, there was a significant decrease in the total number of patient referrals for DM by 12% overall in 2020 compared to 2019 (1,246 *vs* 1,423, respectively) (Figure 4b, *P* < 0.05). However, there was no significant difference in the relative number (i.e. the referral number of DM patients from local clinics to GP, Tongren versus total DM patients seen in clinics) between 2019 and 2020 (Figure 4b, inset, *P >* 0.05).

## Discussion

This study demonstrates that during 2020, the first year of the COVID-19 pandemic, the number of referrals for chronic disease patients between the GP hospital Department and community clinics and, similarly, referrals between the GP service and specialist clinical services, in a tertiary hospital in Shanghai, China, were substantially decreased. This outcome presages a substantial shift in the usual pattern of clinical management of patients with chronic diseases.

We and others have demonstrated that the COVID-19 pandemic has affected the patterns of patient visits (Xu *et al.*, 2021), including a fall in outpatient visits significantly among respiratory patients, probably due to a reduction in the usual respiratory illnesses (Sun *et al.*, 2019). However, acute psychological problems have increased significantly (Xu *et al.*, 2021; Dong and Bouey, 2020; Buhagiar, 2021). It is still unclear how well the patients with chronic conditions have been able to handle the long-term impacts of COVID-19 pandemic.

The patients transferred from the community clinics to Tongren Hospital decreased by ∼20% in 2020, compared to that in 2019, although the proportion of patients transferred as a proportion of total number of patients seen within clinics remained constant. We hypothesise that this was caused by extensive propaganda for viral transmission, thus patients became unwilling to interact within the community (Xu, 2020; Shanghai Municipal Health Commission, 2020). Thus, most of the patients were encouraged to visit local community doctors instead. Secondly, local community doctors also reduced patients’ referral to the GP Unit, Tongren Hospital, unless absolutely necessary (Xu *et al.*, 2021). Similarly, the numbers of patients referred back to the Community clinics was also reduced.

There was no significant difference in the age and sex of the referred patients from the local clinics to Tongren Hospital or *vice versa*.

Following the control of the outbreak of COVID-19 over the latter few months of 2020, the number of patients referred from the local clinics to the GP Department, Tongren Hospital gradually increased, almost reaching a plateau in November 2020. This is probably related to the build-up in the confidence of the general population (Li and Liu, 2020; Pahayahay and Khalili-Mahani, 2020), particularly among those patients with the chronic diseases described here, who had neglected consultations earlier in 2020 (Chudasama *et al.*, 2020). Consequently, the level of patient visits almost returned to the usual level in December 2020, compared to that in December 2019.

A similar pattern of decreased referrals to specialists was observed. Notably, GPs and specialists treat the patients in a cooperative relationship. Thus, during the pandemic, fewer patients were willing to visit major hospitals due to fear of encountering a high pathogen load, unless their condition(s) became unmanageable by the GP in the local clinics during 2020.

When the pattern of referrals for specific chronic diseases was assessed individually, we observed a substantial drop in referrals for hypertension, as a proportion of total consultations for hypertension, but not for DM. In the case of patients with hypertension, the number of patients treated for hypertension in 2020 did not change, in contrast to a significant fall in absolute patient numbers treated by the GP Department in 2020 compared to that in 2019. Overall, patients presumably choose to avoid referral to major hospitals for fear of COVID-19 contamination. However, blood pressure is regulated by two key factors: physical/vascular structures and psychological/emotional stress. The hypertensive patients’ vascular pathophysiological condition could be managed in a reasonably stable manner by the local doctors, but the impact of COVID-19 inevitably increased psychological stress, contributing to poorly controlled hypertension in some patients. Thus, although the total number of patient consultations for hypertension was maintained, referrals were reduced due to fear of COVID-19 contamination within the hospital system. Such an explanation is well supported by previous findings, showing increased numbers of anxious and insomniac patients during the COVID-19 pandemic in 2020 (Xu *et al.*, 2021; Johnson, 2019; Sensoy *et al.*, 2021).

However, we found that the absolute number of DM referrals was reduced in 2020, compared to that of 2019, maybe due to improved clinical skills of the local doctors in handling relatively complicated conditions involving these patients. However, referrals as a proportion of the number of DM patients seen within clinics did not change, probably reflecting a consistent level of diabetic complications occurring irrespective of the COVID-19 pandemic. Of course, the unwillingness of these patients to visit major hospitals also contributed to the reduced number of referrals (Daily, 2020; Eastday.com, 2020; Peng *et al.*, 2020). Notably, exacerbations of DM complications are not usually directly associated with stress, compared to the impact of stress on hypertension.

In conclusion, there were significantly reduced patient referrals from the local community clinics to the GP Unit, Tongren Hospital, and *vice versa*, and a reduction in the referrals from GPs to specialists in Tongren Hospital. There were also reduced referrals for DM patients from local clinics to Tongren Hospital.

Telemedicine has been applied during the pandemic, particularly during the complete lockdown period amid the Omicron event (Xu *et al.*, 2022). Patients with chronically stable conditions were consulted via telemedicine, and medications were delivered by the pharmacy at Tongren Hospital through delegated delivery personnel who had been trained in preventing viral transmission. In any special and critical conditions, these patients were referred to the Emergency Department, at the few special designated COVID-19 hospitals in Shanghai. Such an approach, appreciated by both doctors and patients, could be utilized in future unprepared events.

The strength of this study lies in the unique information our data provides for practitioners during the pandemic, especially for GPs in managing patients under the most challenging conditions. Such insights could be extremely valuable in preparing for any unforeseen future events. However, we acknowledge the limitations of the current study; for example, it was conducted at a single centre within one region (Changning District, Shanghai). Therefore, the data may not fully represent the entire population of Shanghai and/or China. We intend to conduct retrospective studies in the future involving multiple centres across different regions and/or countries.
